# Design of dual-band cold mirrors

**DOI:** 10.1038/s41598-017-15824-5

**Published:** 2017-11-13

**Authors:** Xiaodong Wang, Bo Chen

**Affiliations:** Changchun Institute of Optics, Fine Mechanics and Physics, Chinese Academy of Sciences, Changchun, 130033 China

## Abstract

Dual-band cold mirrors are designed based on third-order of 121.6 nm multilayers, and they are only composed of LaF_3_ and MgF_2_. The designed mirrors have a high reflectance at 121.6 nm and 280 nm, and a low reflectance in visible waveband; they also have a narrow bandwidth of 4 nm at 121.6 nm, and low sidelobe ripples of two stopbands. A broadband antireflection coating and an asymmetrically Gaussian-apodization of thickness-modulated design method are proposed to eliminate sidelobe ripples of the reflection zone.

## Introduction

The Lyman-*α* line (121.6 nm) is emitted from hydrogen element, which is the brightest line in solar chromosphere and transition region of chromosphere and corona. Magnetic field in solar chromosphere can be deciphered by studying Hanle effect in Lyman-*α* line, which is a major challenging task in heliophysics^1–3^. In 2015, the Chromospheric Lyman-Alpha SpectroPolarimeter (CLASP1) sounding rocket was launched by NASA, it first successfully detected linear polarization resulted from scattering process and Hanle effect in 121.6 nm line. In analyzing obtained data, researcher encountered a problem: the scattering polarization was influenced by the local radiation field, which hindered the study of Hanle effect. They argue that an additional spectral region of the Mg II *h* and *k* lines around 280 nm as well as the Lyman-*α* line, is added to be detected, which will contribute to solve this problem^1–3^. Based on this idea, NASA proposes a further project, named as Chromospheric LAyer SpectroPolarimeter (CLASP2). In this project, a dual-band cold mirror is needed, and it will reflect the lines of 121.6 nm and 280 nm, and transmit visible light.

The cold mirror is defined as a narrowband multilayer that reflects targeted wavelengths, and transmits visible light, which was already successfully utilized in CLASP1^3^, Solar Ultraviolet Magnetograph Investigation (SUMI)^4^, and Interface Region Imaging Spectrograph (IRIS)^5^. Targeted wavelength of the reflection zone for the cold mirror in CLASP1 is 121.6 nm, in SUMI is 155 nm and 280 nm, in IRIS is 133–140 nm and 278–283 nm, respectively. Dual-band cold mirrors employed in CLASP2, SUMI, and IRIS are composed of two stacks of multilayer on the same side of the substrate, the first deposited HfO_2_/SiO_2_ multilayer reflects 280 nm line, and the second fabricated LaF_3_/MgF_2_ optical coatings reflects other lines (121.6 nm, 155 nm or 133–140 nm)^4^.

In order to achieve a desirable spectral purity, sidelobe ripples (oscillations in the passbands) of cold mirrors should be as low as possible. Many researchers dedicated their efforts to solving this problem^6–11^. Perilloux systemically proposed a discrete apodization of thickness-modulated design (TMD) method to suppress sidelobe ripples^7–9^. Based on his job, Zhang^10^ and Lyngnes^11^ made some improvements. The apodization of TMD method is derived from Rugate filter design. Rugate filter has a continuous variation of the refractive-index profile^12–14^, while apodization of TMD aims to adjust thickness of layers for achieving targeted spectral response.

In this paper, only one stack of LaF_3_/MgF_2_ optical coating, instead of two stacks, is utilized to design dual-band cold mirrors, and the designed cold mirror has a high reflectance at 121.6 nm and 280 nm, and a low reflectance in visible waveband. A broadband antireflection coating (BAC) and an asymmetrically Gaussian-apodized TMD (AGATMD) are proposed to eliminate sidelobe ripples of the reflection zone.

## Design

**Figure 1:**
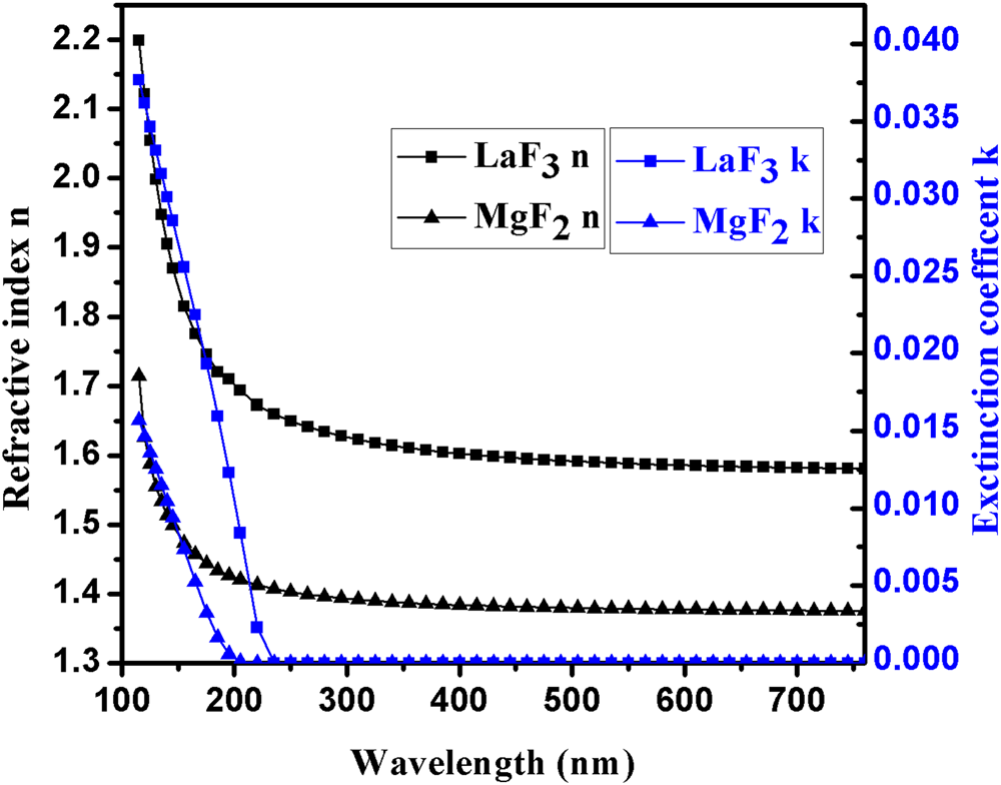
Optical constants of LaF_3_ and MgF_2_ derived by us from characterization of reflectance (10° and 20°) and transmittance (0°) of a single layer (LaF_3_, MgF_2_).

LaF_3_ and MgF_2_ are used in our designed multilayer. Figure 1 shows optical constants of utilized LaF_3_ and MgF_2_, which are derived by us from characterization of reflectance (10° and 20°) and transmittance (0°). The detailed characterization process will be provided in next publication.

Our design includes four parts step by step:

1. Choose an appropriate periodic multilayer by TMD;
2. BAC is added into multilayer to reduce sidelobe ripples of 121.6 nm stopband,
3. Left side of AGATMD of multilayer to suppress oscillations in the passbands of 280 nm reflection zone; Step 2 and 3 are two methods for sidelobe ripples suppression.
4. Optimized by a computer software.

Our designed cold mirror will have three features listed as below:

1. A high reflectance at 121.6 nm and 280 nm, and a low reflectance in visible waveband;
2. Low sidelobe ripples of two stopbands;
3. The bandwidth of 121.6 nm reflectance zone is 4 nm.

### TMD

We use Perilloux’s TMD method to determine initial multilayer structure of this cold mirror. The thickness of discrete layer can be calculated by equation (1)^9^:

1 $$T(L)={T}_{AVG}[1+k\,\cos (2\pi {f}_{m}L)]$$

where *T*(*L*) is optical thickness of the *L*-layer, *T*_*AVG*_ is quarter-wave (QW) optical thickness, *k* is modulation amplitude, and *f*_m_ is modulation frequency.

Here, we first discuss the simplest case of *k* = 0 and *f*_m_ = 0.5, this corresponding multilayer is traditional QW multilayer (*L*/*H* = 1, *L* and *H* is optical thickness of low- and high-index material). It should be noted that *f*_m_ = 0.5 is selected in our discussion because this degenerated TMD case has the lowest number of possible rejection zones between two stopbands. Since we have two targeted wavelengths of 121.6 nm and 280 nm, we should determine the relationship between two targeted wavelengths (order number) and the incidence angle.

The relationship of two targeted wavelengths can be determined by equation (2)^9^:

2 $${\sigma }_{M,N}\approx \frac{{\sigma }_{0}[2N{f}_{m}+(2M-1)]}{1+\frac{1}{2}(\frac{{\rm{\Delta }}{n}_{L}}{{n}_{{L}_{0}}}+\frac{{\rm{\Delta }}{n}_{H}}{{n}_{{H}_{0}}})}$$

where *σ*_*M*,*N*_ is higer-order stopband (wavenumber, nm^−1^), *σ*_0_ is first-order rejection zone, *M* and *N* are integer number, Δ*n*_i_ is refractive index changes of high- and low-index materials due to dispersion of materials, *n*_i0_ is refractive index of high- and low-index materials at fundamental rejection zone. Here, the Δ*n*_i_/*n*_i0_ can be calculated by the data-fitting method in Origin software. We let *σ*_*M*,*N*_ = 1/121.6 nm^−1^, the incidence angle is 0°, and we use equation (2) to determine the value of *M* and *N* by trial and error method. The determined value of *M* and *N* will make *σ*_0_ close to 1/280 nm^−1^. When *M* = 1, *N* = 1 (second order), *σ*_0_ = 1/202 nm^−1^; when *M* = 2, *N* = 0 (third order), *σ*_0_ = 1/300 nm^−1^. Thus, we should select *M* = 2, *N* = 0 because 1/300 nm^−1^ is close to 1/280 nm^−1^. Next, we should make *σ*_0_ equal 1/280 nm^−1^ by increasing the incidence angle.

We introduce the influence of the incidence angle on the position of stopbands into equation (2), which is shown in equation (3):

3 $${\sigma }_{M,N}\approx \frac{{\sigma }_{0}[2N{f}_{m}+(2M-1)]}{1+\frac{1}{2}(\frac{{\rm{\Delta }}{n}_{L}}{{n}_{{L}_{0}}}+\frac{{\rm{\Delta }}{n}_{H}}{{n}_{{H}_{0}}})\frac{1}{\sin \,\alpha }}$$

where *a* is the incidence angle.

It is found that when *a* is assumed to be 45°, the *σ*_0_ equals 1/279 nm^−1^, which is very close to the targeted value of 1/280 nm^−1^.

Thus, 121.6 nm stopband is third-order reflection zone of 280 nm stopband with an incidence angle of 45°. This multilayer structure is our baseline of the design.

It should be noted that the detailed information about *M* and *N* can be found in ref.^9^ and restricted by space reason, we do not provide more explanations.

**Figure 2:**
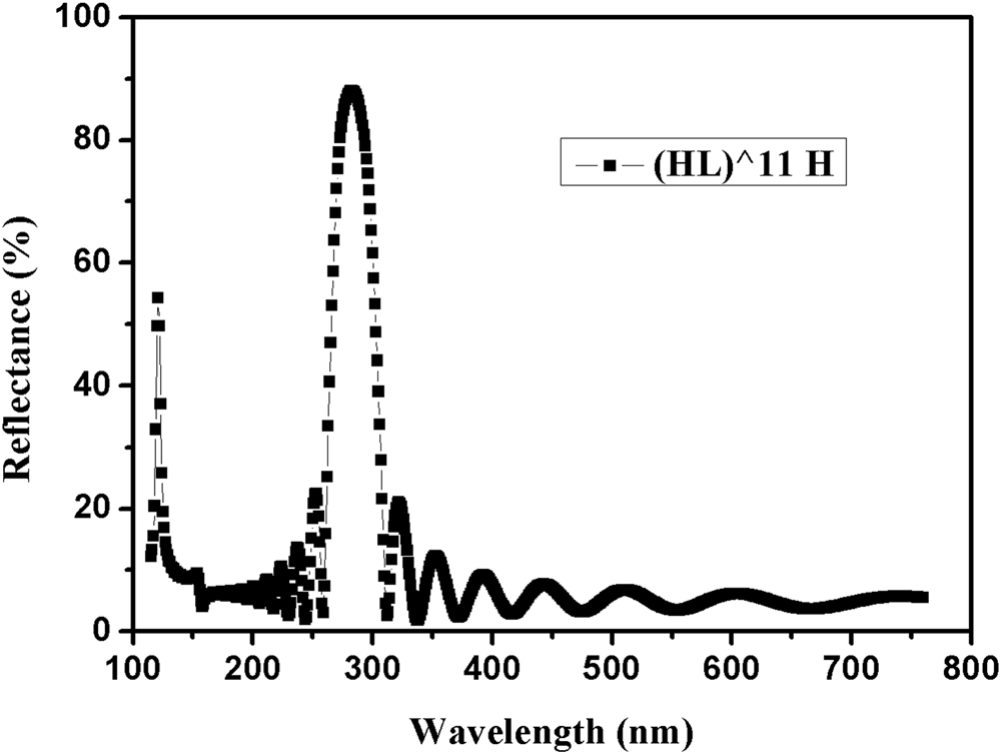
Theoretical reflectance curve of our initial design. The multilayer structure is (HL)^11 H, the incidence is 45°, the substrate is fused silica, backside reflectance is not taken into the calculation, and the reference wavelength is 283 nm.

Figure 2 demonstrates theoretical reflectance curve of our initial design. The multilayer structure is (HL)^11 H, the incidence angle is 45°, the substrate is fused silica, backside reflectance is not taken into the calculation, and the reference wavelength is 283 nm. As shown in Fig. 2, the bandwidth of 121.6 nm stopband is 6 nm, the central wavelength of another reflectance zone is 283 nm, which is slightly deviated from 280 nm, and this can be corrected by next optimization. In addition, sidelobe ripples of two reflection zones are significant, which needs further suppression.

### Sidelobe ripples suppression

The sidelobe ripples of stopbands result from mismatch of optical admittance between multilayer and substrate or air. We first utilize a BAC to suppress sidelobe ripples of 121.6 nm reflection zone, and then employ AGATMD to reduce oscillations in the passbands of 280 nm stopband.

### BAC

**Figure 3:**
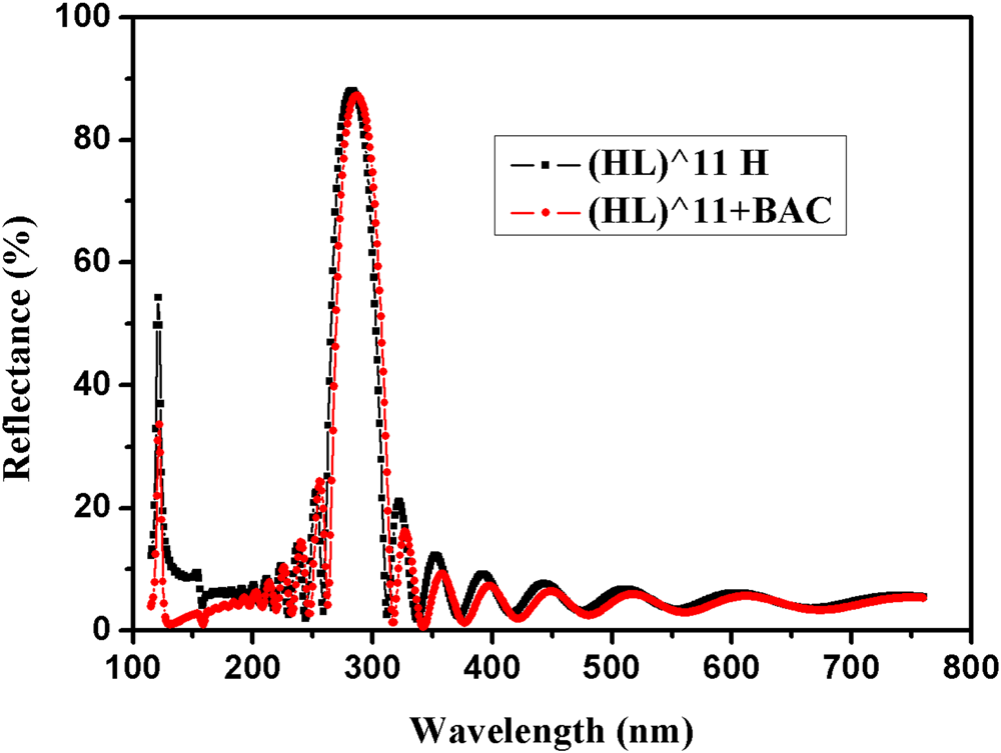
Theoretical reflectance curve of multilayer optimized by BAC optimization. Multilayer structure is (HL)^11 mHmL/2, the value of *m* is determined by the ratio of 121.6 nm to central wavelength, and the central wavelength is 283 nm.

In order to suppress sidelobe ripples of 121.6 nm reflection zone, the 2HL (the reference wavelength is 121.6 nm) layers are inserted between air and multilayer^10^. Figure 3 demonstrates theoretical reflectance curves of multilayer optimized by BAC optimization. Multilayer structure is (HL)^11 mHmL/2, the value of *m* is determined by the ratio of 121.6 nm to central wavelength, and the central wavelength is 283 nm. After BAC optimization, sidelobe ripples of 121.6 nm reflection zone are largely suppressed, and the bandwidth is reduced to be 5 nm. We can see that BAC contributes only to sidelobe ripples reduction of 121.6 nm stopband, and oscillations in the passbands of 280 nm reflection zone are slightly changed, which needs further reduction.

### AGATMD

**Figure 4:**
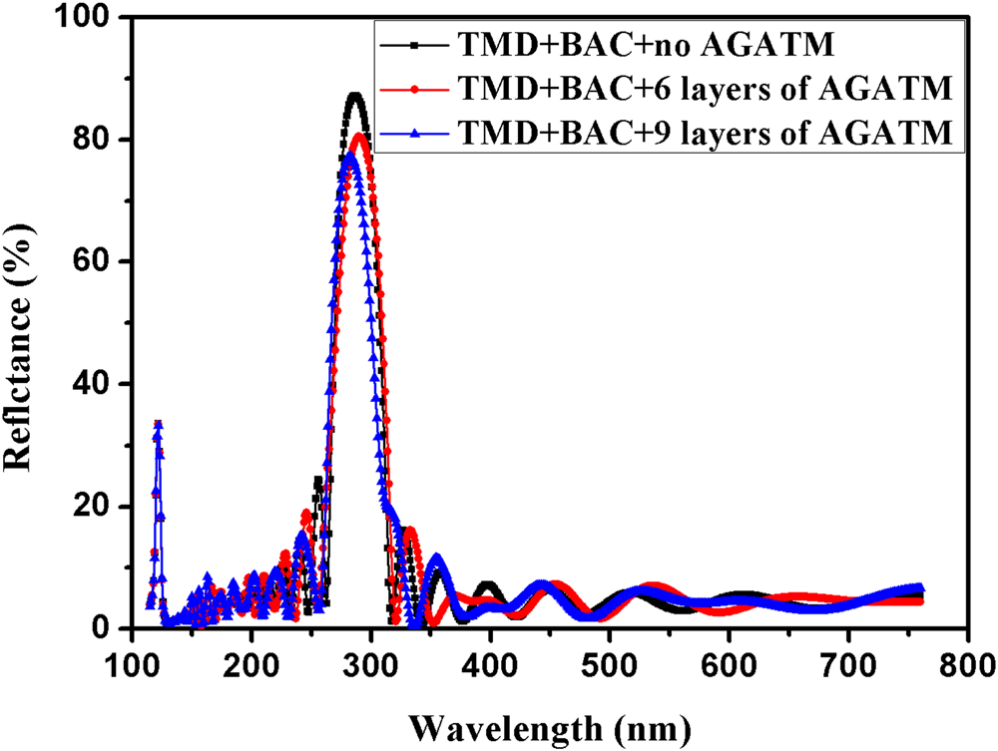
Theoretical reflectance curves of multilayers apodized by 0, 6, and 9 layers of left side of AGATMD, respectively.

In order to reduce sidelobe ripples of the 280 nm stopband, we try to use discrete apodization of TMD method. This method can be described by equation (4)^9^:

4 $$T(L)={T}_{AVG}[1+kA(L)\cos (2\pi {f}_{m}L)]$$

where *A(L)* is amplitude envelope function (sine-wave, linear, quintic function), or Gaussian envelope function. Here, we just discuss Gaussian envelope function, and amplitude envelope function has similar results. Gaussian envelope function is described by equation (5)^11^, and constant *C* is calculated by equation (6)^11^, where *W* is the bandwidth of 280 nm stopband.

5 $$A(L)=\exp (-{(L-{L}_{total})}^{2}/(2{C}^{2}))$$

6 $$C=\frac{W}{\sqrt[2]{2\,\mathrm{ln}(2)}}$$

here *k* does not equal zero, and is assumed to be 2/3, which makes the thickness of first layer close to 10 nm. Since both sides of apodization for TMD multilayer will result in emergence of second-order reflection zone between first- and third stopbands^15^, we choose different layers of AGATMD. It is found that only left side (close to the substrate) of apodization for TMD multilayer results in better spectral results. For brevity, Fig. 4 only provides theoretical reflectance curves of multilayers apodized by 0, 6, and 9 layers of left side of AGATMD, respectively. It is found that the first big sidelobe ripples on the both sides of 280 nm stopband are reduced by left side of AGATMD, 1–9 layers of AGATMD have no influence on spectral performance of 121.6 nm stopband, however, 10 and 11 layers of AGATMD have a bad influence (not drawn in Fig. 4). Spectral performance of designed multilayer is still not ideal, and needs to be optimized by computer software.

### Optimization

**Figure 5:**
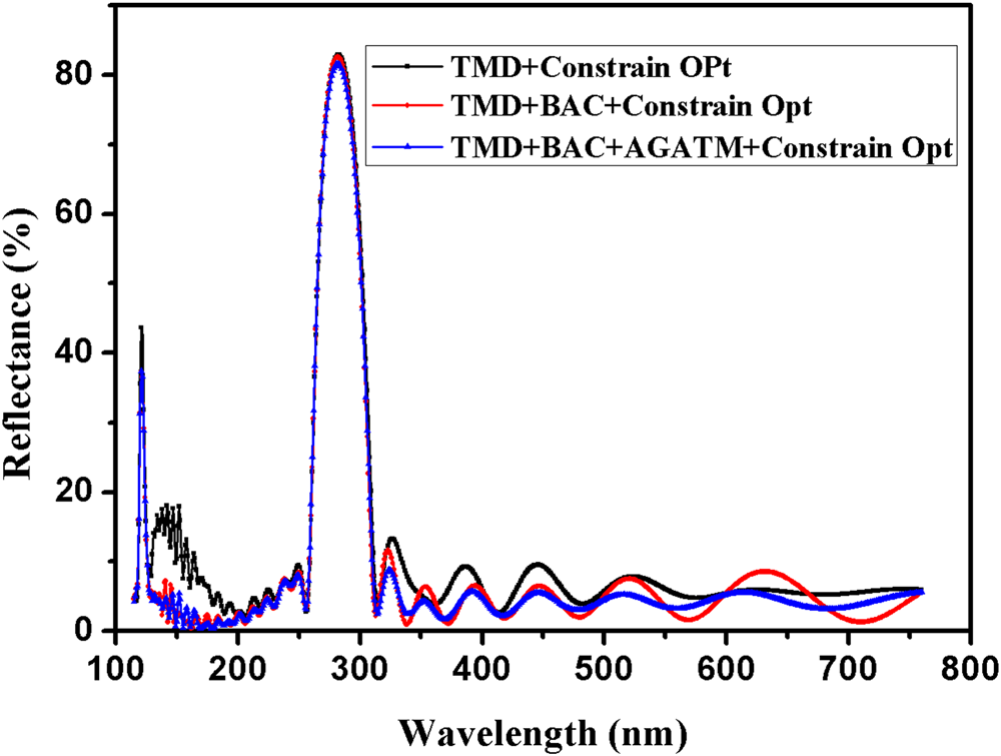
Theoretical reflectance curves of (TMD, TMD + BAC, and TMD + BAC + AGATMD) multilayers optimized by Constrained Optimization Function of OptiLayer software.

**Figure 6:**
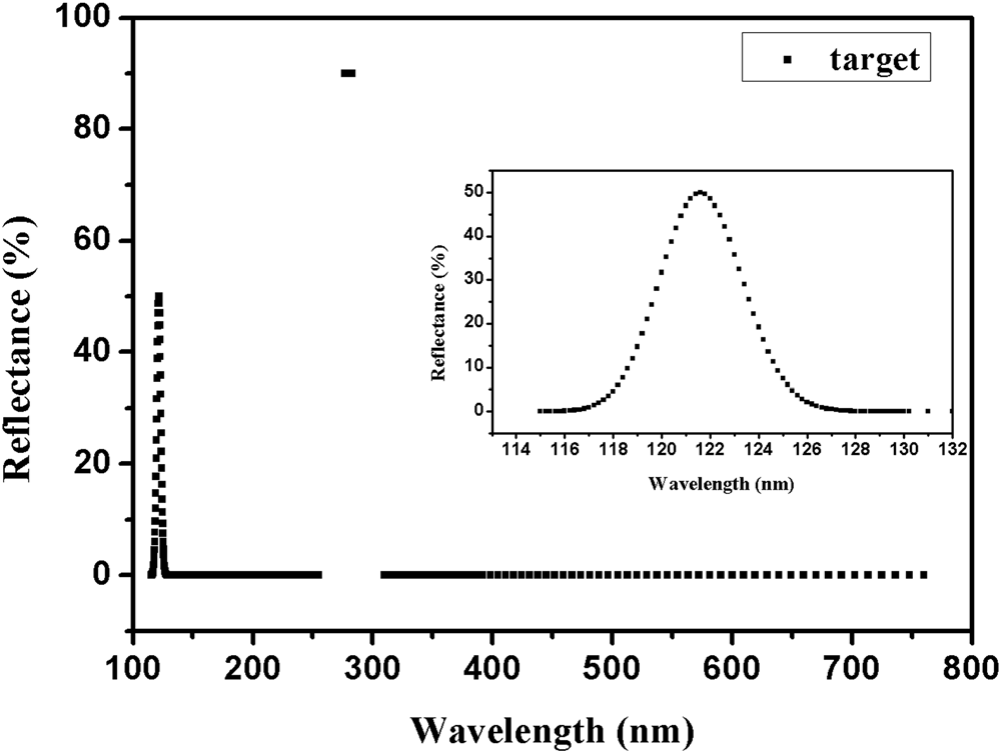
Selected targeted spectral performance in final optimization (Fig. 5) in Optilayer software. The inset is the magnified 121.6 nm stopband with a bandwidth of 4 nm, which is described by a Gaussian type.

We use OptiLayer software^16^ to further optimize the AGATMD of multilayer, and Constrained Optimization Function is utilized. For comparison, QW periodic multilayer, and QW periodic multilayer with the addition of BAC are also optimized by the same Function. All theoretical reflectance curves are shown in Fig. 5. The spectral target in 115–760 nm is shown in Fig. 6, and the inset of Fig. 6 is the magnified 121.6 nm stopband with a bandwidth of 4 nm, which is described by a Gaussian type. It is found that Gaussian type of target curve for 121.6 nm stopband benefits the achievement of the narrow bandwidth of 4 nm.

As shown in Fig. 5, optimization of AGATMD of multilayer reveals the best spectral response; QW periodic multilayer shows the worst. The best designed multilayer has features listed as below:

1. The bandwidth of 121.6 nm stopband is 4 nm,
2. A reflectance of 37.4% at 121.6 nm,
3. A reflectance of 81.4% at 280 nm,
4. Low sidelobe ripples,
5. Low reflectance in visible band.

## Conclusion

We introduce the influence of the incidence angle on the position of reflection zones into traditional Perilloux’s TMD formula, and it is found that when the incidence angle is 45°, 121.6 nm stopband is third-order reflection zone of 280 nm stopband. Thus, our design is based on third-order of 121.6 nm multilayer by TMD. Next, a BAC is applied to reduce oscillations in the passband of 121.6 nm stopband. Then, AGATMD is utilized to suppress sidelobe ripples of 280 nm reflection zone. Finally, Constrained Optimization Function of OptiLayer software is employed to further optimize multilayer, and Gaussian type of target curve for 121.6 nm stopband benefits the achievement of a narrow bandwidth of 4 nm.

Compared with Narukage’s job, our designed cold mirror has a narrower bandwidth at 121.6 nm stopband, and lower sidelobe ripples, and its shortcoming is that the reflectance at 121.6 nm is not high. We utilize only one kind of LaF_3_/MgF_2_ multilayer to achieve targeted spectral response that Narukage employed two kinds of multilayers (LaF_3_/MgF_2_ + SiO_2_/HfO_2_) to realize, our designed multilayer structure is simpler, and easier to be deposited, the spectral performance is also good. Our designed dual-band mirror will contribute most to study magnetic structure of solar chromosphere.
